# *Diaporthe citri*: A Fungal Pathogen Causing Melanose Disease

**DOI:** 10.3390/plants11121600

**Published:** 2022-06-17

**Authors:** Chingchai Chaisiri, Xiangyu Liu, Yang Lin, Chaoxi Luo

**Affiliations:** 1 Key Lab of Horticultural Plant Biology, Ministry of Education, Wuhan 430070, China; chaisiri.ch@webmail.hzau.edu.cn (C.C.); xiangyuliu@webmail.hzau.edu.cn (X.L.); 2 Hubei Key Lab of Plant Pathology, Huazhong Agricultural University, Wuhan 430070, China; yanglin@mail.hzau.edu.cn; 3 College of Plant Science and Technology, Huazhong Agricultural University, Wuhan 430070, China

**Keywords:** citrus, melanose, *Diaporthe citri*, epidemiology, symptomatology

## Abstract

Citrus melanose is a fungal disease caused by *Diaporthe citri* F.A. Wolf. It is found in various citrus-growing locations across the world. The host range of *D. citri* is limited to plants of the *Citrus* genus. The most economically important hosts are *Citrus reticulata* (mandarin), *C. sinensis* (sweet orange), *C. grandis* or *C. maxima* (pumelo), and *C. paradisi* (grapefruit). In the life cycle of *D. citri* throughout the citrus growing season, pycnidia can be seen in abundance on dead branches, especially after rain, with conidia appearing as slimy masses discharged from the dead twigs. Raindrops can transmit conidia to leaves, twigs, and fruits, resulting in disease dispersion throughout small distances. Persistent rains and warm climatic conditions generally favor disease onset and development. The melanose disease causes a decline in fruit quality, which lowers the value of fruits during marketing and exportation. High rainfall areas should avoid planting susceptible varieties. In this article, information about the disease symptoms, history, geographic distribution, epidemiology, impact, and integrated management practices, as well as the pathogen morphology and identification, was reviewed and discussed.

## 1. Introduction

### 1.1. Major Fungal Diseases on Citrus

Several citrus diseases are currently documented in China and around the world. The generally occurring fungal diseases include melanose, gummosis, and stem-end rot caused by *Diaporthe* spp.; branch cankers caused by *Botryosphaeriaceae* [1,2]; scab caused by *Elsinoë* spp. [3,4,5,6,7,8,9]; black rot caused by *Alternaria* spp. [10,11,12,13,14]; greasy leaf spot caused by *Cercosporoid* genus [15,16]; anthracnose caused by *Colletotrichum* spp. [17,18,19,20,21,22,23,24,25]; and blue and green mold caused by *Penicillium* spp. [26,27,28]. Among these fungal diseases, melanose, gummosis, and stem-end rot caused by *Diaporthe* spp. have a significant impact on citrus production [29,30]. At the same time, some *Diaporthe* spp. have also been reported as endophytes and/or saprobes on citrus [29,30,31,32,33,34,35,36,37].

**Figure 1:**
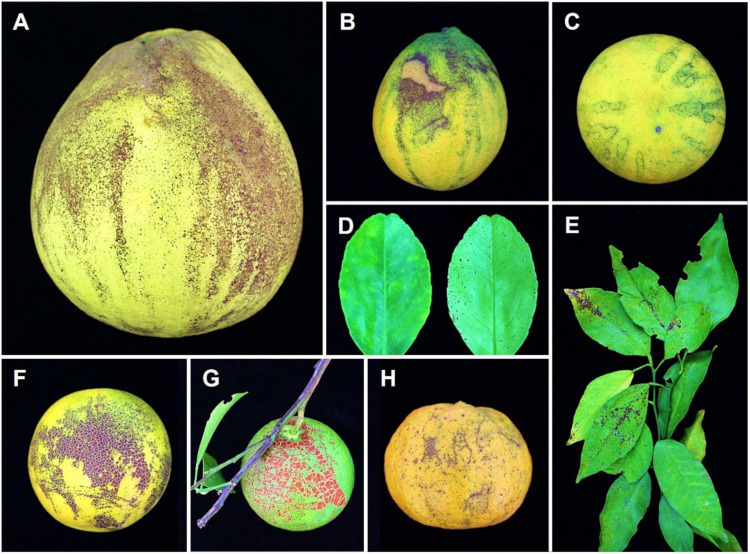
The typical symptoms of melanose disease in the field with different citrus tissue causal agents by *Diaporthe* species: (**A**) pumelo fruit (*C. maxima*) from Chongqing; (**B**,**C**) orange fruits (*C. sinensis*) from Chongqing; (**D**) young orange leaf (*C. sinensis* var. Brasliliensis) from Guizhou; (**E**) mandarin leaf (*Citrus* sp.) from Zhejiang; (**F**) orange fruits (*C. sinensis*) from Chongqing; (**G**) citrus fruit (*C. changshan-huyou*) from Zhejiang; and (**H**) mandarin fruit (*C. reticulata*) from Zhejiang.

Melanose disease was not a major problem in citrus crops prior to the 1990s. However, the accumulation of a large number of dead branches or trees results in an increase in fungal inocula in old citrus orchards worldwide. Currently, melanose has become the major fungal disease of citrus in China, dramatically reducing the commercial value of citrus fruits (Figure 1). *Diaporthe* spp., have been isolated from citrus hosts in many citrus-growing regions of China, e.g., Jiangxi, Zhejiang, Guangxi, Guangdong, Shaanxi, Fujian, Hunan, Chongqing, Yunnan, etc.

### 1.2. Diaporthe Species Associated with Citrus

Previous studies about *Diaporthe* spp. have largely concentrated on species identification, especially the species associated with specific hosts. The molecular taxonomy of the genus *Diaporthe* related to citrus and allied taxa has made great advances in recent years. The phylogenies based on multiple loci provide a more robust and comprehensible taxonomy and nomenclature for *D. citri* and will serve as a starting point for field study by plant pathologists, breeders, and mycologists. Such information may be used to improve disease management and the deployment of citrus cultivars with species-specific and/or broad-spectrum resistance.

All citrus species, including grapefruit, clementine, lemon, lime, mandarin, orange, satsuma, and tangerine, are susceptible to melanose. *Phomopsis citri* was first recorded as a citrus parasitic fungus causing stem-end rot symptoms in Florida, USA [38]. Its teleomorph (sexual stage) is *D. citri* [39]. In addition to *D. citri*, many other *Diaporthe* species were also detected in citrus hosts. They could be pathogens, endophytes, or saprobes on citrus [29,31,40,41,42,43,44,45]. The summary of the global distribution of *Diaporthe* species associated with citrus hosts and their allied genera confirmed with DNA sequences is shown in Table 1.

### 1.3. Identification and Molecular Diagnostics

Citrus melanose is caused by *D. citri*, which belongs to Kingdom Fungi; Ascomycota; Sordariomycetes; Diaporthales; Diaporthaceae; *Diaporthe* [57,58,59,60,61,62,63,64]. The genus of *Diaporthe* was established by Nitschke [65]. *Phomopsis* is the anamorphic (asexual stage) name of *Diaporthe* [38,63,66,67,68,69,70]. The genus *Diaporthe* shows high species diversity; more than 1200 species named “*Diaporthe*” and about 1050 species named “*Phomopsis*” have been recorded in MycoBank lists (http://www.mycobank.org; accessed on 9 June 2021).

#### 1.3.1. Morphological Characteristics

For taxonomy of *Diaporthe* species, morphological characterization based on conidia morphology, fruiting body structure, and culture characteristics has been the basis of this study [71,72,73,74]. On PDA culture medium, mycelium is typically fan-shaped and white in color [75]. Teleomorphic ascomata, which are usually immersed in the substrate erumpent through pseudostromata mostly surrounding the ascomata, have more or less elongated perithecial necks. The pseudostromata are distinct and often delimited by dark lines [76]. The perithecia are circular, flattened at the base, with long black beaks [39,77]. The perithecia generally remain within the plant’s bark but protrude out of the stem surface, which makes them easily visible under a dissecting microscope. Asci are unitunicate and clavate to cylindrical, loosening from the ascogenous cells at an early stage and lying free in the ascocarp. Ascospores are biseriate to uniseriate, and there are two oil droplets or guttulae within each cell, which are fusoid, ellipsoid to cylindrical, septate, straightly constricted at the septum, inequilateral or curved, hyaline, and sometimes with appendages [76,78]. Because ascospores are forcibly ejected from the asci, they become windborne and are responsible for the long-distance spread of the pathogen [39]. Upon finding a suitable substrate, spores may germinate, producing hyphae that quickly become septate mycelium [77].

**Figure 2:**
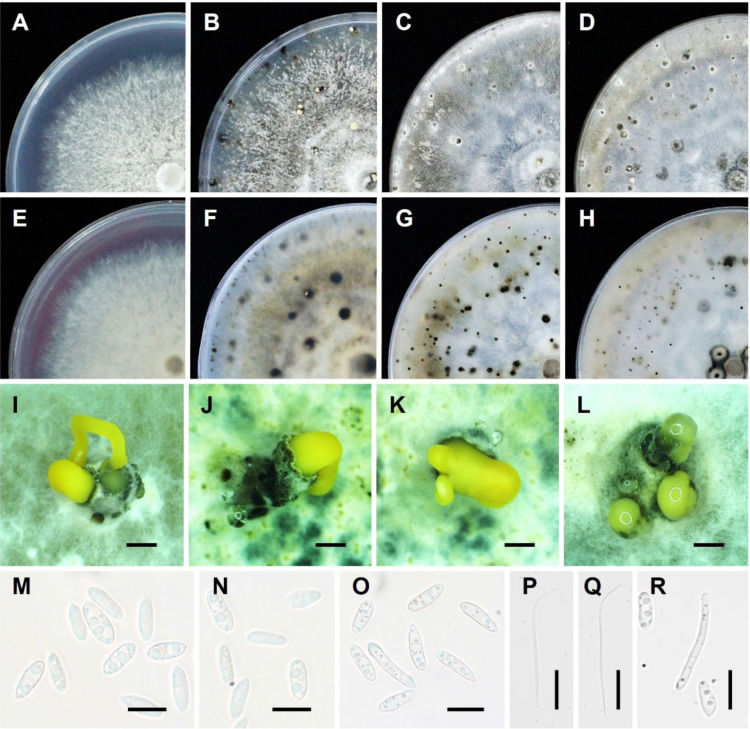
Asexual morphology and cultural characteristics of *D. citri*: (**A**,**E**) culture on PDA medium after 7 days; (**B**,**F**) culture on potato dextrose agar (PDA) medium after 30 days; (**C**,**G**) culture on corn meal agar (CMA) medium after 30 days; (**D**,**H**) culture on oatmeal agar (OMA) medium after 30 days; (**I**–**L**) conidiomata sporulating on PDA medium after 30 days; (**M**–**O**) Alpha conidia; (**P**,**Q**) Beta conidia; and (**R**) Alpha and Gamma conidia. Note: (**A**–**D**) surface and (**E**–**H**) reversed sides of colony culture. Scale bar: (**I**–**L**) = 200 μm; (**M**–**R**) = 10 μm.

The anamorphic state of this fungus is the most important stage for the disease cycle. The pycnidia (asexual fruiting bodies) of *D*. *citri* are scattered on the substratum and are dark in color, ovoid, thick-walled, and erumpent. Conidiophores are hyaline and branched, and occasionally, they are short and 1–2 septate. Conidiogenous cells were phialidic, hyaline, and slightly tapering toward the apex [30,37]. Generally, they are multiseptate and filiform with enteroblastic and monophiladic conidiogenesis [79,80]. It may produce three types of hyaline, non-septate conidia, namely, alpha, beta conidia [81], as well as an intermediate between these two conidial types, namely, gamma conidia [82,83,84]. The alpha conidia are functional, aseptate, single-celled, hyaline, fusiform, and usually biguttulate but sometimes lack guttula (lipid drop) or have more guttulae. The beta conidia tend to be produced in older pycnidia and are also aseptate, hyaline, long, slender, rod-shaped structures. They may be filiform and straight, but more often they are hooked at one end, lack guttula, and do not germinate [85]. The gamma conidia are hyaline, multiguttulate, fusiform to subcylindrical, with an acute or rounded apex, while the base is sometimes truncate [73,82,83,84,86,87]. The asexual morphology and cultural characteristics of *D. citri* are shown in Figure 2.

#### 1.3.2. Molecular Identification

Currently, four nuclear genome sequences of *D. citri* have been deposited in GenBank with the accession numbers JACTAD000000000, JADAZQ000000000, JADAZP000000000, and JADAZO000000000 for strains NFHF-8-4, ZJUD2, ZJUD14, and Q7, respectively [88,89]. The genome assembly sizes of ZJUD2 (59.5 Mp) and ZJUD14 (52.0 Mp) were relatively shorter, while NFHF-8-4 and Q7 contained longer assembly size (more than 63 Mp) [88,89].

Taylor et al. [90] proposed genealogical concordance phylogenetic species recognition (GCPSR), which compares individual gene sequences to find inconsistencies, and it has been shown to be very useful in defining species boundaries in morphologically conserved fungi [91]. Although each cluster in combined trees is usually considered a separate lineage, the common approach of concatenating sequenced data to delimit species without using the GCPSR principle overestimates the real diversity of species placement [91,92,93,94,95,96]. Since the widespread use of DNA sequences [35], genus *Diaporthe* species identification has progressed beyond host association and morphological characterization [73,81]. The *Diaporthe* genus is commonly represented by using traditional molecular barcoding for fungal species identification based on nuclear ribosomal internal transcribed spacer regions (ITS) [70,97,98,99]. As a result, some *Diaporthe* species have been reported to be perplexing, with contradictory findings when only the ITS sequence is used to produce a phylogenetic tree [35,67,99,100,101,102].

According to previous studies, multi-locus phylogenetic analysis has been proved more efficient to identify isolates at the species level [29,35,102,103,104,105]. Several loci, including large subunit of the ribosomal DNA (LSU), intergenic spacers of the ribosomal DNA (IGS), ITS, translation elongation factor 1-α gene (*TEF1-α*), ß-tubulin gene (*TUB2*), histone 3 gene (*HIS3*), calmodulin gene (*CAL*), actin gene (*ACT*), DNA-lyase gene (*APN2*), 60S ribosomal protein L37 gene (*FG1093*), and mating type genes (*MAT-1-1-1* and *MAT-1-2-1*), are demonstrated as efficient tools to determine *Diaporthe* species. Even molecular sequences are already being used to identify species and rebuild phylogenies, complete genome sequences for *Diaporthe* species are still in the future. Currently, the most frequently used molecular loci in this genus are the ITS, *TEF1-α*, *TUB2*, *HIS3*, and *CAL* [35,58,98,104]. Among them, *TEF1-α* is the most efficient tool in resolving the phylogenetic signal of the *D. eres* species complex [101,106]. Similarly, the highly variable *TEF1-α* was also shown to be the most efficient locus in distinguishing *Diaporthe* species [99,101,104,106,107]. Although the ITS region showed the relatively limited delimitation of *Diaporthe* species in phylogenetic analyses, it is still informative and should not be excluded from concatenation analysis of multi-locus DNA sequences [58,94,98,104]. A summary of universal and species-specific primers used for species determination within the *Diaporthe* genus is shown in Table 2.

#### 1.3.3. Molecular Diagnosis

A conventional species-specific PCR method has been developed to distinguish *D. citri* from other *Diaporthe* species [32,46,117]. The PCR-based technique showed outstanding specificity and sensitivity, indicating that it may be used to effectively detect *D. citri* in practice. Effective PCR with citrus tissues infected by *D. citri*, as well as modern PCR, isothermal amplification, or any technique that is fast, low-cost, and accurate for alternative detection of certain diseases, should be developed, because such methods may also be applied for phytosanitary detection in plant quarantine.

#### 1.3.4. Genetic Populations

Although *D. citri* and other *Diaporthe* infections are well-known, information about their diversity, population genetics, reproductive methods, and pathogenicity is limited [125,126]. Our understanding of the infection process, host range, and fungicide resistance of *D. citri* would improve if we understood its population genetics in nature. Such information is also useful for making long-term management strategies for this disease [127,128,129]. In China, the population genetics of *D. citri* was analyzed by using polymorphic simple sequence repeat (SSR) markers and the mating type idiomorphs. The majority of the analyzed samples came from southern China, including Fujian, Zhejiang, Jiangxi, Hunan, and Guizhou provinces. It was shown that alleles at the 14 SSR loci were not substantially different from linkage equilibrium, and most subpopulations exhibited equal frequencies of the two mating types. The findings suggest that teleomorphic reproduction is important in *D. citri* populations in southern China, and the ascospores seem to be a major contributor to citrus disease [46]. The presence of significant genetic differences among different geographical populations, however, does not eliminate the possibility of migration. Closely related strains were detected from many geographically diverse regions. They also found signs of genetic mixing between two extremely distinct genetic populations. These findings imply that *D. citri* populations are evolving, which might be accelerated by either increasing human impacts through frequent citrus seedling exchange or by global climate change [46].

## 2. Epidemiology, Life Cycle, and Symptomatology

Citrus melanose is caused by *D. citri*, which attacks foliage, fruits, and twigs when they are immature. Since mature tissues are more immune to pathogen attack, the first 8 to 9 weeks of the citrus growing season are the most vulnerable to pathogen attack. Melanose signs can differ depending on the severity of the infection. At the end of the susceptibility cycle, the flyspeck melanose symptoms appear [130,131].

The fungal inocula can be scattered over a wide range since ascospores are released forcefully and can be spread over a long distance. *D. citri* is primarily a saprophyte that feeds on and receives its nutrition from dead wood [44,132]. Perithecia and pycnidia are only found on dead and dying twigs and fruits showing stem-end rot. The conidia provided by pycnidia are the primary source of inoculum [133]. Ascospores are ejected forcibly and play a significant role in long-distance dispersal [132]. As a result of the widespread dissemination of a vast number of ascospores, the number of cases of infection is rising [134]. When ascospores or conidia of *Diaporthe* land on the surface of a plant, the disease will be triggered. Pathogens thrive in dry environments with temperatures ranging from 17 to 35 °C [44].

The germination of spores requires approximately 10 to 24 h of moisture, depending on the temperature [44,134], and the germination and formation of a germ tube takes 36 to 48 h [133]. After that, the citrus melanose pathogen directly penetrates the cuticle layer tissue and infects the plant.

**Figure 3:**
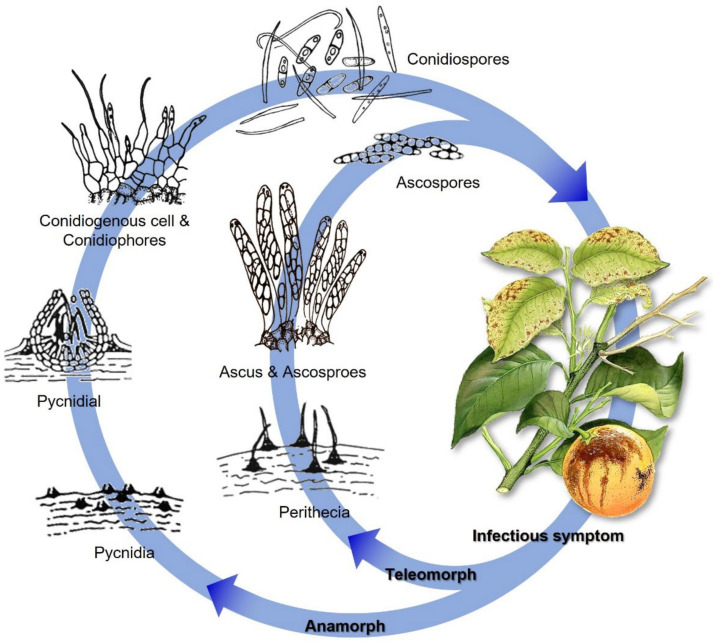
Representative *Diaporthe* disease cycle: melanose disease cycle on citrus caused by *D. citri*. Revised and redrawn from Burnett [135], Timmer et al. [75], and Udayanga et al. [63].

*D. citri* could overwinter on debris, e.g., mummy fruits, dead stems, branches, and dry leaves. Perithecia could form on debris next year. Ascospores are produced in proportion to the amount of dead wood present in a canopy. These spores contribute slightly to the disease severity of an orchard, but they are carried by the wind and spread across long distances. Conidia, developed in mature pycnidia, can continuously infect citrus during the growing season. Conidia can be dispersed to nearby citrus trees with raindrops, which most probably cause the majority of fruit infections (Figure 3). Nevertheless, conidia can also be transmitted through the air over long distances when rainfall is scarce.

Symptoms appear as discrete small, sunken, brown spots about one week after infection, which later become raised and filled with reddish-brown gum. The leaf pustules are initially surrounded by a yellow halo. Diseased areas regreen later and create corky pustules. On fruits, pustules can grow relatively large and can crack, creating a pattern of mudcake. The severity of the disease is determined mainly by the amount of inoculum-bearing dead wood in the canopy of the tree and the duration of the wetting period following rainfall or sprinkler irrigation. Wet, rainy conditions, especially when rain showers occur late in the day, and fruits staying continuously wet on warm nights are conducive to infection.

## 3. Geographic Distribution and Host Associations

**Figure 4:**
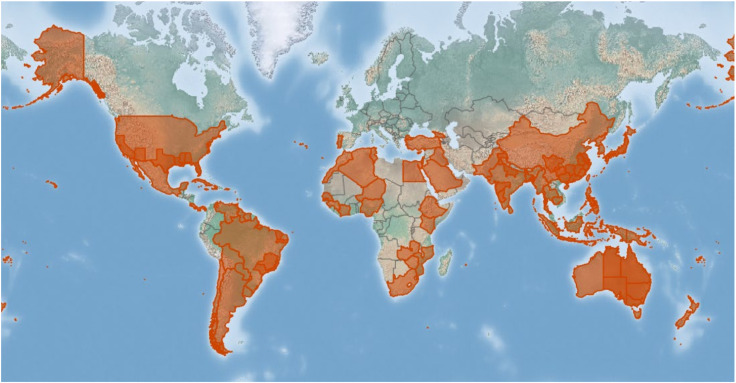
A global geographic distribution map of *D. citri* associated with the *citrus*-host plant is available on the CABI database.

The USDA’s Agricultural Research Service’s Systematic Mycology and Microbiology Laboratory (SMML database: https://nt.ars-grin.gov/fungaldatabases/, accessed on 10 December 2021) and the Centre for Agriculture and Bioscience International (CABI database: https://www.cabi.org/isc/, accessed on 15 January 2022) obtained some information about *D. citri*, including the geographic distribution and host associations. According to the SMML and CABI databases, *D. citri* has been discovered on citrus hosts and related species all over the world. The *D. citri* is the most predominant species in the *Diaporthe* genus, which occurs widely in citrus-growing countries, e.g., China, Philippines, Japan, Korea, Thailand, Myanmar, Cambodia, Fiji, Mauritius, United States, Mexico, Haiti, Cuba, Dominican Republic, Panama, Puerto Rico, Venezuela, Trinidad and Tobago, Brazil, Cyprus, Portugal (Azores Islands), New Zealand, Niue, Samoa, Tonga, Cook Islands, Cote d’Ivoire, and Zimbabwe, which has also been summarized previously [32]. A global geographic distribution map of *D. citri* associated with the citrus hosts is available on the CABI database (accessed and last modified on 16 November 2021) and is shown in Figure 4. The green disease-free areas may mean that no data is in the CABI database, but this does not necessarily mean that the disease is absent.

## 4. Main Management Approaches of Melanose Disease

The yield is almost unaffected by melanose disease, and the juice processing is unaffected as well. However, the quality of the fruit for marketing and exportation suffers the consequences. In order to avoid poor quality and fruit deterioration caused by citrus melanose, integrated management practices should be implemented. Integrated pest management (IPM) is now largely recognized as the most effective way to protect plants. Its ultimate objective will be to maintain pest populations below economically injurious levels without or just with minimal pesticides. Although IPM must rely on pesticides currently, minimizing chemical inputs while maintaining crop quality at an economically viable level is a basic requirement for plant protection. To achieve this goal, it is critical to understand the disease epidemiology at various points in time while performing pest control [132,136].

Currently, no resistance cultivars are available for melanose control in practice. The removal of dead wood to reduce the pressure of melanose fungus is both time-consuming and labor-intensive. Nevertheless, pruning dead branches should be performed on a regular basis. Proper pruning enhances air circulation within the canopy of the plant, keeping it dry and reducing opportunities for pathogens to survive and cause infections. It will also improve the effectiveness of fungicide infiltration into the foliage [43,137]. Furthermore, avoid planting sensitive citrus cultivars or species in high-rainfall zones, such as sweet orange, grapefruit, and pumelo [137,138]. Other management practices, such as citrus plantations in low-rainfall and sunny zones, should be implemented. Interplanting citrus with non-susceptible hosts is also a feasible measure [137,139].

### 4.1. Chemical Control

Application of fungicides is still the most commonly used method to control melanose disease on citrus. Many fungicides have been tested for melanose control. Copper is a protective compound, which forms a layer on the surface of plant tissue, e.g., fruit, protecting it from infection. The gap in the protective copper layer, however, grows larger as the fruit grows and expands. If conditions are favorable for the pathogen infection, the copper layer needs to be renewed through another spray. The melanose fungus stored in dead wood is slightly affected by copper spraying. The use of copper fungicides before flowering will not reduce infection. A copper fungicide must be applied on the fruit surface to provide efficient melanose control. In the case of serious infection in late summer, additional protectant spray should be applied [140]. Applications of pyraclostrobin to the spring flush growth of citrus trees are much more efficient for controlling melanose, scab, and Alternaria brown spot than those of famoxadone or copper hydroxide [44,141,142,143,144]. Bushong and Timmer [145] demonstrated that azoxystrobin was a highly effective preventative spray for melanose, whereas benomyl and fenbuconazole were not. As post-infection treatments for melanose, none of the fungicides are successful. In Japan, dithianon and mancozeb were used to spray alternately from June to August to control this disease [146]. In Pakistan, five chemicals were tested at recommended doses, including penflufen, copper hydroxide, tebuconazole plus trifloxystrobin, and difenoconazole, for controlling melanose disease. When used as a protectant, copper hydroxide was found to be the most effective for the management of citrus melanose [147]. Whereas Anwar et al. [148] evaluated six different fungicides for citrus melanose control, the use of mancozeb led to a significant inhibition of fungal growth. Similarly, several chemicals, including mancozeb and fenbuconazole, were found to be effective in controlling citrus melanose in China and other countries [40,43,148,149,150].

### 4.2. Biological Control

Although chemical control plays an important role in managing plant diseases, overuse of chemical pesticides has raised severe issues about food contamination, environmental pollution, and phytotoxicity. Biocontrol is a viable option as it is friendly to the environment. Biological control of plant diseases with antagonistic bacteria is a viable alternative to chemical control. Many antagonistic bacteria are known to play important roles in the sustainability of natural ecosystems, and some of them can be employed as inoculants to stimulate plant growth and resistance.

For melanose control, more and more biocontrol candidates have been developed, e.g., *Burkholderia gladioli*: TRH423-3, MRL408-3, *Pseudomonas pudia*: THJ609-3, and *P. fluorescens*: TRH415-2, and selected for their antifungal effectiveness against *D. citri* using dual-culture testing. Disease suppression was observed after pretreatment with the rhizobacterial strains, with varying degrees of protection rates for each rhizobacterial strain. Following the pathogen inoculation, subsequent treatment with the rhizobacterial strains also enhanced protection rates. The rhizobacterial strains might be especially useful in organic citrus production where chemicals are strictly forbidden [151]. Similarly, pre-treatment with *P. putida* strain THJ609-3 resulted in a decreased disease incidence. When the infection behaviors of *D. citri* and necrosis deposits on plant tissues were examined using a fluorescent microscope, it was shown that the process of disease development was reduced after being treated with the bacterial strain, especially the conidia germination rates, which were significantly lower after being pretreated with the strain THJ609-3. Furthermore, morphological abnormalities of the germ tubes were also observed. These results pointed to the bacterial-direct antifungal action on the leaf surfaces as a potential cause of disease reduction [152]. *Thiobacillus* species were used to generate bio-sulfur, which was investigated as an alternative to managing citrus melanose. It was found that melanose disease severity was lower on bio-sulfur pretreated citrus leaves than on untreated leaves, suggesting that bio-sulfur might be applied as an environmentally friendly alternative to control citrus melanose [153]. *Bacillus velezensis* CE 100, an effective biocontrol agent, has been used to control *D. citri*. In dual culture plates, *D. citri* mycelial growth was significantly suppressed by strain CE 100, suggesting that some volatile substances inhibited the growth of *D. citri*. It was also observed that the bacterial culture filtrate (BCF) of strain CE 100 inhibited *D. citri* growth. Microscopic examination indicated that BCF had a substantial impact on the pathogen hyphal shape, most probably the result of numerous cell-wall disintegrating enzymes and metabolites generated by strain CE 100. Interestingly, *D. citri* conidial germination was decreased by approximately 80% when 50% BCF of strain CE 100 was used [154].

## 5. Conclusions

In this paper, the history of citrus melanose, pathogen morphology, molecular identification, population studies, epidemiology of disease symptoms and life cycle, global distribution, and integrated disease management are documented. At present, there are no cases of plants bred or engineered specifically for resistance to diseases caused by *D. citri*. Does the teleomorph *D. citri* have higher opportunities for surviving on different hosts? Does genetic recombination play an important role in survival or in variability in this species? Are teleomorph ascospores spread differently from anamorphic conidia? Do ascospores and conidia infect citrus tissues in the same way? Which defense reactions occur in infected plants? At what level is the pathogen *D. citri* recognized by the plant? Are signal cascades of defensive reactions known? These are some of the questions about pathogen epidemiology that still need to be answered as they directly impact disease management. However, more understanding of the molecular mechanisms that confer virulence on *D. citri* is helpful in the development of alternative disease management strategies, especially when it is urgent to develop environmentally friendly approaches or tools to maintain the plant health in the future.

## Figures and Tables

**Table 1:** Summary of the global distribution of *Diaporthe* species associated with *Citrus* hosts and their allied genera confirmed with DNA sequences.

*Diaporthe* Species	*Citrus* Host and Allied Genera	Locality Distribution	Symptom/Tissue	Reference(s)
*D. apiculatum*	*Citrus grandis* cv. Tomentosa	China	non-symptom/twig	[34]
*D. aquatica*	*C. grandis* cv. Tomentosa	China	non-symptom/fruit	[34]
*D. arecae*	*C. grandis*	China	non-symptom/twig, leaf	[31]
	*C. limon*	China	non-symptom/branch	[31]
	*C. reticulata*	China	non-symptom/branch, twig	[31]
	*C. sinensis*	China	non-symptom/branch, twig	[31]
	*C. sinensis*	Suriname	Decaying/fruit	[35]
	*C. unshiu*	China	non-symptom/twig	[31]
*D. biconispora*	*C. grandis*	China	non-symptom/branch	[31]
	*C. sinensis*	China	non-symptom/branch	[31]
	*Fortunella margarita*	China	non-symptom/branch	[31]
*D. biguttulata*	*C. limon*	China	non-symptom/branch	[31]
*D. citri*	*C. reticulata*	China	Melanose, stem-end rot, dead wood/fruit, leaf	[30,31,37,46]
	*C. reticulata*	New Zealand	N.A./stem	[37]
	*C. reticulata*	Portugal (Azores)	Blight/shoot	[47]
	*C. reticulata* cv. Nanfengmiju	China	Melanose/fruit, leaf, twig	[32]
	*C. sinensis*	Brazil	N.A./fruit	[37]
	*C. sinensis*	China	Melanose/twig, leaf	[32,46,48]
	*C. sinensis*	USA, Florida	Stem-end rot/fruit	[30]
	*C. unshiu*	China	non-symptom/twig	[31]
	*C. unshiu* var. Juwadeun	Korea	N.A./fruit	[37]
	*Citrus* sp.	USA, Florida	N.A./leaf	[37]
*D. citriasiana*	*C. grandis* cv. Shatianyou	China	Anonymous spot/leaf	[31]
	*C. reticulata* cv. Nanfengmiju	China	Melanose-like/leaf	[32]
	*C. sinensis*	China	Melanose-like/leaf	[32]
	*C. unshiu*	China	Dead wood, non-symptom /branch, leaf	[30,31]
*D. citrichinensis*	*C. grandis*	China	non-symptom/branch	[31]
	*C. unshiu*	China	Dead wood, scab/branch, leaf	[30,31]
	*Fortunella margarita*	China	non-symptom/branch	[31]
*D. cytosporella*	*C. limon*	Spain	N.A./fruit	[37]
	*C. limonia*	Italy	N.A.	[37]
	*C. sinensis*	USA, California	N.A./twig	[37]
*D. discoidispora*	*C. reticulata* cv. Nanfengmiju	China	Melanose-like/fruit, leaf	[32]
	*C. sinensis*	China	non-symptom/twig	[30,31]
	*C. unshiu*	China	non-symptom/twig	[31]
*D. endocitricola*	*C. grandis* cv. Tomentosa	China	non-symptom/fruit	[34]
*D. endophytica*	*C. unshiu*	China	Scab/leaf	[31]
*D. eres*	*C. reticulata* cv. Nanfengmiju	China	Melanose-like/twig, fruit, leaf	[32]
	*C. unshiu*	China	Non-symptom/twig	[31]
	*Fortunella margarita*	China	Non-symptom/branch	[31]
	*Citrus* sp.	China	Non-symptom/branch, fruit	[31]
*D. foeniculina*	*C. aurantiifolia*	Greece	Blight, canker/shoot, branch	[49]
	*C. aurantiifolia-limon*	Greece	Blight, canker/shoot, branch	[49]
	*C. bergamia*	Greece	Canker/branch	[29]
	*C. japonica*	Malta	Dieback/twig	[29]
	*C. latifolia*	USA, California	N.A./truck	[37]
	*C. limon*	Greece	Blight, canker/shoot, branch	[29,49]
	*C. limon*	Italy	Canker/trunk	[29]
	*C. limon*	Malta	Canker/trunk	[29]
	*C. limon*	New Zealand	N.A.	[37]
	*C. limon*	Portugal	Dieback/twig	[29]
	*C. limon*	Spain	Dieback/twig	[29,37]
	*C. limon*	Turkey	Rot/fruit	[50]
	*C. limon*	USA, California	N.A./branch	[29,37]
	*C. limon*	Lebanon	Blight/shoot	[42]
	*C. maxima*	Greece	Canker/branch	[29]
	*C. maxima*	Italy	Canker/branch	[29]
	*C. medica*	Greece	Blight, canker/shoot, branch	[49]
	*C. mitis*	Italy	Canker, dieback/branch, twig	[29]
	*C. paradisi*	Italy	Canker/branch	[29]
	*C. paradisi*	Malta	Canker/trunk	[29]
	*C. paradisi*	Portugal	Canker/branch	[29]
	*C. reticulata*	Greece	Dieback/twig	[29]
	*C. reticulata*	Italy	Dieback/twig	[29]
	*C. reticulata*	Spain	Dieback/twig	[29]
	*C. sinensis*	Iran	Non-symptom/leaf	[51]
	*C. sinensis*	Italy	Canker/branch, trunk	[29]
	*C. sinensis*	Malta	Canker/branch	[29]
	*C. sinensis*	Portugal	Canker, dieback/branch, twig	[29]
	*Microcitrus australasica*	Italy	Dieback/twig	[29]
	*Poncirus trifoliate × C. paradisi*	Greece	Blight, canker/shoot, branch	[49]
*D. foeniculina* (*D. baccae*)	*C. limon*	Italy	Blight, canker/shoot, branch	[29]
	*C. paradisi*	Italy	Canker/branch	[29]
	*C. reticulata*	Italy	Canker/trunk	[29]
	*C. sinensis*	Italy	Canker, dieback/trunk, twig	[29]
*D. guangdongensis*	*C. grandis* cv. Tomentosa	China	non-symptom/fruit	[34]
*D. hongkongensis*	*C. grandis*	China	Non-symptom/twig	[31]
	*C. reticulata*	China	Scab/leaf	[31]
	*C. reticulata* cv. Nanfengmiju	China	Non-symptom/twig	[31]
	*C. sinensis*	China	Non-symptom/twig	[31]
	*C. unshiu*	China	Scab/leaf	[31]
*D. infertilis*	*C. sinensis*	Suriname	Decaying/fruit	[29,35]
*D. limonicola*	*C. grandis* cv. Tomentosa	China	non-symptom/fruit	[34]
	*C. limon*	Malta	Canker/branch, trunk	[29]
*D. masirevicii*	*C. grandis* cv. Tomentosa	China	non-symptom/fruit, twig	[34]
*D. melitensis*	*C. limon*	Malta	Canker/branch	[29]
*D. multigutullata*	*C. grandis*	China	Non-symptom/branch	[31]
	*C. maxima*	China	Symptomatic/branches	[48]
*D. novem*	*C. aurantiifolia*	Italy	Dieback/twig	[29]
	*C. japonica*	Italy	Dieback/twig	[29]
*D. ovalispora*	*C. limon*	China	non-symptom/twig	[31]
*D. passifloricola*	*C. grandis* cv. Tomentosa	China	non-symptom/fruit, twig	[34]
	*C. reticulata* cv. Nanfengmiju	China	Stem-end rot/fruit	[52]
*D. perseae*	*C. grandis* cv. Tomentosa	China	non-symptom/leaf	[34]
*D. phaseolorum*	*C. limon*	Cameroon	non-symptom/leaf	[41]
*D. sennae*	*C. grandis* cv. Tomentosa	China	non-symptom/fruit	[34]
*D. siamensis*	*C. sinensis*	China	Stem-end rot/fruit	[53]
*D. sojae*	*C. limon*	China	Non-symptom/twig	[31]
	*C. limon*	Cameroon	non-symptom/leaf	[41]
	*C. reticulata*	China	Non-symptom/twig	[31]
	*C. reticulata* cv. Nanfengmiju	China	Melanose-like, scab/twig, fruit, leaf	[31,32]
	*C. unshiu*	China	Non-symptom/twig	[31]
*D. subclavata*	*C. grandis* cv. Shatianyou	China	Unidentified symptom/fruit	[31]
	*C. unshiu*	China	Scab/leaf	[31]
*D. taoicola*	*C. sinensis*	China	Stem-end rot/fruit	[53]
*D. unshiuensis*	*C. reticulata* cv. Nanfengmiju	China	Melanose-like/fruit, twig	[32]
	*C. sinensis*	China	Melanose-like/twig, leaf	[32]
	*C. unshiu*	China	Unidentified symptom/fruit	[31]
	*Fortunella margarita*	China	Non-symptom/branch	[31]
*Diaporthe* sp.	*C. aurantium*	Taiwan	non-symptom/N.A.	[54]
	*C. limon*	India	Dieback/shoot, branch	[55]
	*C. limon*	Cameroon	non-symptom/leaf	[41]
	*C. reticulata*	Iran	non-symptom/N.A.	[56]
	*Fortunella margarita*	China	Non-symptom/branch	[31]

N.A.: not available.

**Table 2:** Summary of published universal primers and species-specific primers used for species determination within *Diaporthe* spp.

Gene/Locus ^1^	Primer Name	Primer Sequences (5′ to 3′)	Reference
*ACT*	ACT-512F	ATGTGCAAGGCCGGTTTCGC	[108]
	ACT-783R	TACGAGTCCTTCTGGCCCAT	[108]
	ACT878R	ATCTTCTCC ATGTCGTCCCAG	[37]
*APN2*	apn2fw2	GCMATGTTYGAMATYCTGGAG	[101]
	apn2rw2	CTTGGTCTCCCAGCAGGTGAAC	[101]
*CAL*	CAL-228F	GAGTTCAAGGAGGCCTTCTCCC	[108]
	CAL-737R	CATCTTCTGGCCATCATGG	[108]
	CL1	GARTWCAAGGAGGCCTTCTC	[109]
	CL2A	TTTTTGCATCATGAGTTGGAC	[109]
	CAL563F	GACAAATCA CCACCAARGAGC	[37]
*FG1093*	FG1093 E1F1	GCGCCACAMCAAGWCSCACRC	[110]
	FG1093 E3R1	TTCTBCGCTTGGCCTTCTCRS	[110]
*GAPDH*	Gpd1-LM	ATTGGCCGCATCGTCTTCCGCAA	[111]
	Gpd2-LM	CCCACTCGTTGTCGTACCA	[111]
*HIS3*	CYLH3F	AGGTCCACTGGTGGCAAG	[112]
	H3-1b	GCGGGCGAGCTGGATGTCCTT	[113]
IGS	IGS-12a	AGTCTGTGGATTAGTGGCCG	[114]
	NS1R	GAGACAAGCATATGACTAC	[114]
ITS	ITS1	TCCGTAGGTGAACCTGCGG	[115]
	ITS-1F	CTTGGTCATTTAGAGGAAGTAA	[116]
	ITS4	TCCTCCGCTTATTGATATGC	[115]
	DcitriF	GTTTAACTACTGCGCTCGGGGTCCTG	[117]
	DcitriR	CTTACTGTTGCCTCGGCGCAGG	[117]
LSU	LSU1Fd	GRATCAGGTAGGRATACCCG	[118]
	LR5	TCCTGAGGGAAACTTCG	[119]
*MAT1-1-1*	MAT1-1-1FW	GCAAMIGTKTIKACTCACA	[99]
	MAT1-1-1RV	GTCTMTGACCARGACCATG	[99]
	MAT1 141F	GGTCAAGAAGAAGAAGTCC	[120]
*MAT1-2-1*	MAT1-2-1FW	GCCCKCCYAAYCCATTCATC	[99]
	MAT1-2-1RV	TTGACYTCAGAAGACTTGCGTG	[99]
	MAT2 188F	CCAGCTCCATCACAAC	[120]
*MS204*	MS204 E1F1	AAGGGCACCCTGGAGGGCCAC	[110]
	MS204 E5R1	GATGGTGACGGYGTTGATGTA	[110]
SSU	NMS1	CAGCAGTGAGGAATATTGGTCAATG	[121]
	NMS2	GCGGATCATCGAATTAAATAACAT	[121]
*TEF-α*	EF1-728F	CATCGAGAAGTTCGAGAAGG	[108]
	EF1-986R	TACTTGAAGGAACCCTTACC	[108]
	EF-2	GGARGTACCAGTSATCATGTT	[122]
*TUB2*	Bt2a	GGTAACCAAATCGGTGCTGCTTTC	[113]
	Bt2b	ACCCTCAGTGTAGTGACCCTTGGC	[113]
	TUBDcitri-F1	CCATTTGACCATCTGCAACAT	[32]
	TUBD-R1	CCTTGGCCCAGTTGTTTCC	[32]
	Dc-F	CCCTCGAGGCATCATTAC	[46]
	Dc-R	ATGTTGCAGATGGTCAAATGG	[46]
	Tub2FD	GTBCACCTYCARACCGGYCARTG	[123]
	T22	TCTGGATGTTGTTGGGAATCC	[124]
	T1	AACATGCGTGAGATTGTAAGT	[124]

^1^ *ACT*: actin gene, *APN2*: DNA-lyase gene, *CAL*: calmodulin gene, *FG1093*: 60s ribosomal protein L37 gene, *GAPDH*: glyceraldehyde-3-phosphate dehydrogenase, *HIS3*: histone 3 gene, IGS: intergenic spacers of the ribosomal DNA, ITS: nuclear ribosomal internal transcribed spacer, LSU: large subunit of the ribosomal DNA, *MAT-1-1-1*/*MAT-1-2-1*: mating type genes, *MS204*: guanine nucleotide-binding protein subunit beta-like protein gene, SSU: small subunit 18S ribosomal RNA, *TEF1-α*: translation elongation factor 1-α gene, and *TUB2*: ß-tubulin gene.

## Data Availability

Not applicable.
